# Model exchange with the NeuroML model database

**DOI:** 10.1186/1471-2202-15-S1-P171

**Published:** 2014-07-21

**Authors:** Sharon M Crook, Suzanne Dietrich

**Affiliations:** 1 School of Mathematical and Statistical Sciences, Arizona State University, Tempe, AZ 85287, USA; 2 School of Life Sciences, Arizona State University, Tempe, AZ 85287, USA; 3 School of Mathematical and Natural Sciences, Arizona State University, Phoenix, Arizona 85609, USA

## 

The Neural Open Markup Language project, NeuroML, is an international, collaborative initiative to develop a language for describing and sharing complex, multiscale neuron and neuronal network models [1]. The project focuses on the key objects that need to be exchanged among software applications used by computational neuroscientists. Examples of these objects include descriptions of neuronal morphology, the dynamics of ion channels and synaptic mechanisms, and the connectivity patterns of networks of model neurons. This modular approach brings additional benefits: not only can entire models be published and exchanged in this format, but each individual object or component, such as a specific calcium channel or excitatory synapse, can be shared and re-implemented in a different model. The use of a standardized description language based on XML also facilitates the development of tools that promote simulator interoperability.

The NeuroML Model Database (NeuroML-db.org) is a relational database that provides a means for exchanging multiscale NeuroML model descriptions and their components. There are several existing, curated model databases that include neuroscience models in multiple formats such as ModelDB [2] and the BioModels Database [3], which provide excellent resources for sharing diverse, published neuroscience models in multiple formats. In contrast, NeuroML-db is more focused, including only NeuroML models. This emphasis allows the database design and search to take advantage of this specific format, and in particular, allows for efficient searches over sub-components of models. In addition, the NeuroML Model Database can be used to search over public models developed as part of the Open Source Brain (OSB) initiative, which provides a software infrastructure for the collaborative development and evaluation of models [4]. NeuroML is the preferred model description format for OSB, and OSB models that are described using NeuroML version 2.0 can be flagged for automatic inclusion in NeuroML-db.

An overarching design goal for the database is to provide a simple keyword search interface. Because many search terms involve terminology from neuroscience, we complement a metadata-based keyword search with a mechanism that can provide information about the biological meaning of the keywords, where this semantic information is available through an existing ontology for neuroscience called NeuroLex [5]. Through implicit query reformulation, this semantic information is used to expand the search results. For example, if the query is a single brain region, all cell and network models for cells and networks from that region and its sub-regions are provided to the user.
